# Does Metabolic Syndrome Affect the Incidence and Prognosis of Sudden Sensorineural Hearing Loss?

**DOI:** 10.3390/life12070930

**Published:** 2022-06-21

**Authors:** Joong Su Park, Seung Ho Kim, Ikhee Kim, Hantai Kim, Ji Hyun Kim, Jong Bin Lee

**Affiliations:** 1 Department of Otorhinolaryngology-Head and Neck Surgery, Konyang University College of Medicine, Daejeon 35365, Korea; sujp1004@kyuh.ac.kr (J.S.P.); 400867@kyuh.ac.kr (S.H.K.); kwingh7.7@gmail.com (I.K.); noto.hantai@gmail.com (H.K.); 2 Department of Pediatrics, Dongguk University Hospital, Goyang 10326, Korea; eogurdl@gmail.com; 3 Myunggok Medical Research Institute, Konyang University College of Medicine, Daejeon 35365, Korea

**Keywords:** metabolic syndrome, sudden sensorineural hearing loss, microvascular injury, case-control study

## Abstract

Earlier studies reported that the occurrence of sudden sensorineural hearing loss (SSNHL) is associated with chronic metabolic disorders such as hypertension, diabetes, and hyperlipidemia. Instead of focusing on the relationship between SSNHL and each metabolic disorder, this study aimed to identify the association with metabolic syndrome as a whole, including either prehypertension or prediabetes. As a case-control study, we reviewed 239 patients who experienced SSNHL, and compared them with the same number of healthy subjects (*N* = 478). Metabolic syndrome-related variables of SSNHL patients were compared to those of healthy control subjects. In addition, patients with SSNHL were classified into two subgroups: the first subgroup showed improvement in hearing (‘response group’), and the second did not present significant improvement (‘non-response group’). Metabolic syndrome was diagnosed according to the US National Heart, Lung, and Blood Institute’s National Cholesterol Education Program Adult Treatment Panel III criteria. The risk for SSNHL was 4.3 times higher in patients with metabolic syndrome compared with patients without the syndrome (95% confidence interval, 1.98 to 9.33), even after adjusting for variables that showed significant between-group differences. The likelihood of being unresponsive to treatment was higher in those with metabolic syndrome (1.21 to 3.93; adjusted odds ratio = 2.18), and when the initial hearing loss pattern on a pure-tone audiometry was high tone or flat. Metabolic syndrome appears to be an independent risk factor for SSNHL and, simultaneously, a predictor of poor prognosis.

## 1. Introduction

Sudden sensorineural hearing loss (SSNHL) occurs suddenly without a clear cause. It is a frightening, otorhinolaryngological disorder requiring treatment within 2–3 days. The annual prevalence rate is 5–10 per 100,000 people in the US and >10 per 100,000 in Korea [1,2]. Typically, SSNHL is diagnosed if hearing loss occurs suddenly in <3 days and hearing is reduced >30 dB in three consecutive frequencies on separate pure-tone audiometry tests performed within a 3 day duration [3]. Viral infection, vascular disease, autoimmune disease, genetic disorder, and metabolic disease are possible etiologies. However, 85–90% of patients are idiopathic, and the causes are identified only in <5% of them [4].

Metabolic syndrome is a cluster of metabolic abnormalities that includes abdominal obesity, hypertension, dyslipidemia, and high blood sugar level, initially labeled as Syndrome X by Gerald Reaven in 1988 [5]. The American Heart Association and World Health Organization have established diagnostic criteria for metabolic syndrome. However, the commonly used criteria are the US National Heart, Lung, and Blood Institute’s (NHLBI) National Cholesterol Education Program Adult Treatment Panel III Guidelines (NCEP-ATP III) [6], specifying five risk factors: (1) ≥150 mg/dL hyperlipidemia–triglycerides, (2) <50 mg/dL high-density lipoprotein cholesterol (HDL-C) for women and <40 mg/dL for men, (3) ≥130 mmHg hypertension systolic blood pressure and ≥85 mmHg diastolic blood pressure, (4) ≥100 mg/dL high blood sugar-fasting glucose, and (5) ≥90 cm abdominal obesity—waist circumference (value varies regionally and racially) for Korean men and ≥85 cm for Korean women [6,7]. A diagnosis of metabolic syndrome is made when ≥3 of the risk factors exist. Metabolic syndrome leads to vascular endothelial dysfunction, increased body inflammation, and adipose tissue accumulation, which is a risk factor for cardiovascular and cerebrovascular disease (such as diabetes, hypertension, chronic kidney disease, stroke, myocardial infarction, and angina pectoris) [8].

Recently, ongoing SSNHL pathophysiology research has uncovered links between the risk factors for cardiovascular and cerebrovascular diseases and the occurrence and prognosis of SSNHL [9]. Disorders such as diabetes, hypertension, obesity, and dyslipidemia are pathological conditions causing microvascular injury and complications. Thus, they may have also a close association with cochlear ischemia, a widely accepted cause of SSNHL [10].

Accordingly, this study hypothesized that metabolic syndrome influences the occurrence and recovery from SSNHL, given that the pathology of the disease is a microvascular injury. The primary objective of this case-control study was to examine the incidence of metabolic syndrome in patients with SSNHL and in healthy subjects with the same size. The second objective was to investigate the effect of metabolic syndrome on the prognosis of SSNHL.

## 2. Materials and Methods

### 2.1. Subjects

The institutional review board of Konyang University Hospital approved this retrospective cohort study. The subjects were patients who visited the Department of Otorhinolaryngology at Konyang University Hospital between January 2013 and December 2016 due to a sudden loss of hearing and were diagnosed with SSNHL based on pure-tone and speech audiometry tests. All patients who met the diagnostic criteria for SSNHL were admitted for inpatient treatment, requesting their participation in this study. Before the initiation of treatment, blood test (including lipid panel), past medical history, and blood pressure and anthropometric measurements (including waist circumference) were performed to diagnose the presence of metabolic syndrome. Eleven patients were excluded in this step; their loss of hearing was because of other causes, such as brain lesion (i.e., vestibular schwannoma, cerebral infarction, or cerebral hemorrhage), Meniere’s disease, multiple sclerosis, head trauma, exposure to noise, and past ear surgery. Finally, 239 subjects were enrolled as the patient group, who were followed up successfully for 3 months.

The control group (*n* = 239 as the same number) was randomly selected from individuals with normal hearing, and who underwent the same tests, including anthropometric measurements, necessary for making a diagnosis of metabolic syndrome. The patient and control groups were then matched according to age and sex.

### 2.2. Treatment of the Patient Group

An MRI of the temporal lobe was performed during the hospital stay to screen vestibular schwannoma or congenital hearing loss, in addition to impedance audiometry, otoacoustic emission, and auditory brainstem response tests. All subjects in the patient group were treated with steroid combination therapy: intravenous high-dose dexamethasone (0.14 mg/kg) and intratympanic dexamethasone injection daily for 5 days. During the stay, they were also treated with acyclovir (5 mg/kg, 3 times per day) and gingko biloba (80 mg, twice per day) intravenously for 5 days. Patients with unstable blood sugar levels were controlled using subcutaneous sliding scale insulin injection. After the steroid combination therapy for 5 days, patients were discharged and received peroral steroid for tapering during next 5 days.

**Figure 1:**
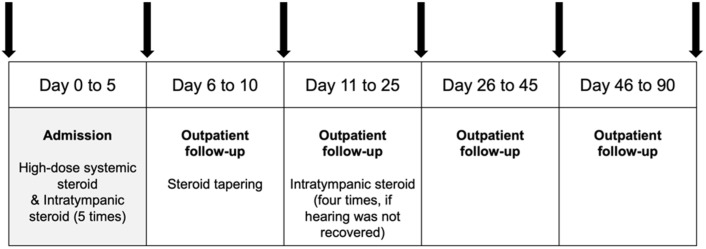
Flowsheet for treatment of sudden sensorineural hearing loss patients. An arrow indicates when the audiometry was performed.

The patient group had repeated pure-tone and speech audiometry tests on the fifth and the eleventh days. If hearing was not entirely recovered by then, patients were given 4 times intratympanic dexamethasone injection over 2 weeks. The fourth pure-tone and speech audiometry tests were performed on the twenty-fifth day. Additional audiometry was performed on days 45 and 90, and the threshold on the 90th day was regarded as the final hearing level (Figure 1).

### 2.3. Definition of Metabolic Syndrome

Metabolic syndrome was diagnosed with NCEP-ATP II, with Asian-specific waist circumference criteria (Table 1) [7]. A definite diagnosis was made if the patient had ≥3 risk factors.

### 2.4. Assessment of Hearing

Hearing was repeatedly assessed on the day of the hospital visit, at days 5, 11, 25, 45, and 90 after the beginning of the treatment. The hearing loss pattern was classified as one of 4 categories (low tone, high tone, flat, and total) by Sheehy’s criteria [11]. Severity of the loss was classified as mild if the mean threshold on 6 frequencies (250, 500, 1000, 2000, 4000, and 8000 Hz) was lower than 41 dB, moderate if 41–70 dB, severe if 71–90 dB, and profound if worse than 90 dB.

Hearing recovery was evaluated by comparing the thresholds of pure-tone audiometry af the first day with those of the 90th day. Final recovery level was determined by Siegel’s criteria (Table 2) [12]. To analyze the factors affecting the treatment outcome, patients that showed complete, partial, or slight recovery were classified as the response subgroup and those without recovery the non-response subgroup.

### 2.5. Statistical Analyses

Statistical analyses used SPSS Statistics for Windows, version 18.0 (SPSS Inc., Chicago, IL, USA). Continuous variables are reported as mean ± standard deviation and categorical variables in frequency and proportion. The independent *t*-test, paired *t*-test, and *Chi*-square test were performed to compare the demographic and audiological characteristics between the groups and identify factors affecting the responsiveness of the treatment. Multivariate logistic regression was conducted to compute adjusted odds ratios and 95% confidence intervals of factors affecting the treatment outcome. In all analyses, *p* < 0.05 indicated statistical significance.

## 3. Results

### 3.1. Characteristics of the Patient and Control Groups

The base characteristics of the patient and control groups are in Table 3. The mean age of the patient group was 52.3 ± 16.3 years old, and the mean age of the control group was 54.5 ± 15.0 years old. Due to the matched study design, sex and age were not statistically different between the groups.

The patient group scored significantly higher than the control group on risk factors for metabolic syndrome (waist circumference, fasting blood glucose, triglycerides, systolic blood pressure, and the history of diabetes). However, a difference was not found in HDL-C, diastolic blood pressure, and past medical history of hypertension.

### 3.2. Metabolic Syndrome and Sudden Sensorineural Hearing Loss

The results of the five risk factors for metabolic syndrome for both groups are in Table 4. The prevalence of metabolic syndrome was significantly higher in the SSNHL patient group than in the control group, 41.4% versus 19.3%. Multivariate logistic regression analysis was performed to adjust the risk of SSNHL by the variables. Using those significant variables in the multivariate analysis, only the ‘metabolic syndrome’ was identified as a risk factor for SSNHL (aOR = 4.30, 95% confidence interval [1.98 to 9.33], *p* < 0.001).

### 3.3. The Severity of Hearing Loss and Post-Treatment Recovery

The SSNHL group was divided into the response subgroup, who showed hearing improvement by ≥15 dB regardless of the hearing loss pattern and their recovered hearing ability was either complete recovery, partial recovery, or slight improvement. The non-response subgroup improved <15 dB, or their recovered hearing threshold was <75 dB. The response subgroup was made up of 153 (64.0%) patients, and the non-response subgroup included 86 (36.0%) (Table 5 and Table 6).

The mean hearing threshold at the time of the initial visit was 71.4 ± 20.9 dB in the response subgroup and 78.5 ± 24.9 dB in the non-response group (*p* = 0.019). The final hearing threshold after the treatment was 34.3 ± 21.5 dB in the response subgroup and 75.6 ± 26.9 dB in the non-response subgroup (*p* < 0.001). The hearing threshold improved by 40.2 ± 18.2 dB in the response and 4.1 ± 12.8 dB in the non-response subgroups (*p* < 0.001). Regarding the severity and pattern of hearing difficulty at the initial hospital visit (Table 6), low tone and flat patterns were characteristics in the response subgroup, whereas high tone and total loss patterns were characteristics in the non-response subgroup (*p* = 0.004). Additionally, the proportion of severe hearing loss was high in the response subgroup, while profound hearing loss was more prevalent in the non-response subgroup (*p* = 0.006).

### 3.4. Prognostic Factors of Sudden Sensorineural Hearing Loss

The response and non-response subgroups were significantly different in age, sex, height, and involved ear side; however, there were no statistical differences in medical histories of hypertension, diabetes, and dyslipidemia (Table 5). Furthermore, the subgroups showed group differences in duration (between the onset of hearing loss and the initial hospital visit) and the presence of the co-occurrence of vertigo, but not of tinnitus. Regarding the prevalence of metabolic syndrome, 52 (34.0%) in the response subgroup and 47 (54.7%) in the non-response group had metabolic syndrome, showing a significant between-group difference (*p* = 0.002). However, the analyses on the individual elements of metabolic syndrome showed that the subgroups were similar to each other, except for HDL-C (20.9% in the response group versus 40.7% in the non-response group; *p* = 0.005).

Multivariate logistic regression analysis was performed to identify prognostic factors. First, to identify prognostic factors among the significantly different independent variables between subgroups, we underwent the univariate analyses for each factor (age, sex, height, lesion side, duration from the onset to the visit, presence of vertigo and metabolic syndrome, and severity and pattern of hearing loss) (Table 7). The likelihood for a patient to be unresponsive to treatment increased with delayed treatment (aOR = 1.102, 95% confidence interval [1.033 to 1.175], *p* = 0.003) and the presence of metabolic syndrome (aOR = 2.182, 95% confidence interval [1.211 to 3.932], *p* = 0.009). Additionally, treatment outcome was more likely non-responsive if it was high tone (aOR = 4.100, 95% confidence interval [1.157 to 14.534], *p* = 0.029) or total loss (aOR = 4.600, 95% confidence interval [1.320 to 16.026], *p* = 0.017).

## 4. Discussion

SSNHL is a disease in the ear requiring appropriate treatment within 2-3 days. There are a variety of theories on the pathophysiology, effective treatments, and prognostic factors; however, these have no steady consensus, and many studies are still underway. Viral infections, the autoimmune response, trauma, vascular disease, metabolic problems, and so on are regarded as the cause of SSNHL, but the etiology is not clear yet. The cochlea is a peripheral organ receiving blood only through the labyrinthine artery, one of the anterior inferior cerebellar artery branches originating from the basal artery. Due to weak collateral blood circulation, the organ has a structural problem in that its function is easily compromised, even with temporary ischemia. Thus, the possibility that SSNHL occurs because of an interruption in the blood flow to the cochlea is widely speculated [13]. Because an interrupted blood flow has been suggested as the pathophysiology, several studies reported that, related to the blood flow interruption, patients with cardiovascular risk factors such as dyslipidemia, diabetes, hypertension, and smoking have an unfavorable prognosis in SSNHL [14,15,16].

The pathogenesis of metabolic syndrome is also not clear. Pro-inflammatory cytokines, such as tumor necrosis factor alpha (TNF-α) or interleukin-6 (IL-6), are elevated in the serum of metabolic syndrome patients, and an increased level of pro-inflammatory cytokines may play an essential role in the pathogenesis of the syndrome [17]. Research showing that these cytokines facilitate inflammatory responses in blood vessels, inducing atherosclerosis, support the hypothesis. Substances such as TNF-α might induce insulin resistance and increase systolic blood pressure by generating hydrogen peroxide [18,19]. Therefore, it may affect the blood flow in the micro-vessels and inflammation in the cochlea.

In Korea, according to the National Health and Nutrition Examination Survey (NHANES) conducted in 2001, 2005, and 2007, the prevalence of metabolic syndrome was 29.2%, 30.3%, and 31.3%, respectively [20]. In 1998, the rate was 19.9% in men and 23.7% in women; the increase is noteworthy. Currently, the prevalence of metabolic syndrome in Korea is similar to or even higher than western countries [20,21]. A study pointed out that the increasing prevalence of metabolic syndrome in Korea might be due to dyslipidemia and abdominal obesity, which had been much lower in the past [20]. Similarly, the number of SSNHL patients was 49,894 in 2009 and reached 61,892 in 2013, with a steady increase at an annual rate of 2–8%, as with metabolic syndrome [2]. Given that metabolic syndrome is a risk factor for SSNHL, this increasing number of people with the syndrome would raise the incidence of SSNHL in the future. Meanwhile, in our study, the prevalence of the syndrome was only 19.3% in the control group, lower than the national incidence. This was because when we collected the control group subjects, age and sex were matched with the SSNHL group. Compared to the aforementioned national cross-section study, more subjects of a younger age were included, and thus, the relatively lower incidence of the syndrome was identified.

In this study, the prevalence of hypertension was 26.7% in the SSNHL and 19.7% in the control groups. The 2013 Statistics Korea (KOSTAT) survey found that the prevalence among adults ≥30 years old in Korea was 27.3%, which is different from the current study’s control group. This may be due to the same reason as with the prevalence rate of metabolic syndrome. On the other hand, diabetes was 17.6% in the SSNHL group and 11.3% in the control group, also close to the KOSTAT results (11.0%).

Among many prognostic factors associated with SSNHL, the initial hearing level at the first visit is regarded as significant [22]. The more severe the hearing loss, the poorer the prognosis; the prognosis is more favorable in patients showing a low- to mid-frequency loss. The present study also suggests that the likelihood for a patient to be unresponsive to treatment is significantly higher when the hearing loss pattern was total (aOR = 4.6) or high tone (aOR = 4.1). In addition, delayed treatment (longer duration to the first hospital visit) was significant (aOR = 1.1). However, the presence of vertigo was significant in the univariate analysis but not in the multivariate analysis.

Hypertension, diabetes, and dyslipidemia are known to have an association with the outcome of SSNHL [2,9,10], but there is little research on the relationship between metabolic syndrome, as a disease cluster, and SSNHL. Because the diagnostic criteria for metabolic syndrome include those diseases, this study might seem to be undifferentiated from the previous literature. However, the diagnostic criteria for metabolic syndrome also include prehypertension and prediabetes; thus, patients diagnosed with neither hypertension nor diabetes may be diagnosed with metabolic syndrome if abdominal obesity or dyslipidemia is present.

Chien et al., reported that metabolic syndrome increased the risk of SSNHL by 3.5 times [23]. This study identified not only that SSNHL patients were more susceptible to metabolic syndrome but also that metabolic syndrome was associated with the prognosis of SSNHL. The key findings of the study were that metabolic syndrome, which can impair cochlear perfusion, may affect the treatment responsiveness in SSNHL. Therefore, patients diagnosed with SSNHL should be preemptively evaluated for the presence of metabolic syndrome; if metabolic syndrome is diagnosed, a patient counseling should be performed with a consideration of the possibility of poorer outcomes due to the syndrome.

Note that the diagnosis of metabolic syndrome includes either prehypertension (systolic BP 130–139 or diastolic BP 85–89 mmHg according to the classification criteria used in Korea; 15 in the SSNHL and 9 in the control groups in this study population) or prediabetes (fasting glucose 100–125; 150 in the SSNHL and 77 in the control groups). Usually, patients with prehypertension and prediabetes do not use medications for their management. However, considering that metabolic syndrome is a risk factor for the prognosis of SSNHL, any preemptive drug use at the pre-stage might improve the outcome. There was a study that co-administration with nimodipine, which has an antihypertensive effect, and systemic steroid deduced the better prognosis in SSNHL [24]; it may also be the effect of the preemptive use of the medication. Therefore, in future studies, it would be necessary to identify the effectiveness of the early use of either antihypertensive medication or the lowering of glucose medication.

## 5. Conclusions

Recently, several studies have reported the relationship between SSNHL and other medical factors such as diabetes, hypertension, and other vascular diseases; however, little study has been conducted on the association between SSNHL and metabolic syndrome. In this study, metabolic syndrome was more common in SSNHL patients, and patients with metabolic syndrome showed poorer responsiveness to the treatment than those without the syndrome. The comorbidity of metabolic syndrome should be regarded to influence the prognosis of SSNHL.

## Figures and Tables

**Table 1:** Criteria for clinical diagnosis of metabolic syndrome by National Cholesterol Education Program Adult Treatment Panel III (NCEP-ATP III).

Elevated waist circumference ^1^	≥90 cm in men ≥85 cm in women
Elevated triglycerides	≥150 mg/dL (1.7 mmol/L) or on drug treatment for elevated triglyceride
Reduced HDL-C	≤40 mg/dL (1.03 mmol/L) in men ≤50 mg/dL (1.3 mmol/L) in women or on drug treatment for reduced HDL-C
Elevated blood pressure	≥130 mmHg systolic pressure ≥85 mmHg diastolic pressure or on antihypertensive drug treatment
Elevated fasting glucose	≥100 mg/dL or on drug treatment for elevated glucose

Abbreviation: HDL-C, high-density lipoprotein cholesterol. ^1^ The criteria of elevated waist circumference are quoted by the criteria of Korean society of the study of obesity.

**Table 2:** The Siegel’s criteria of hearing recovery.

Type	Hearing Recovery
Complete recovery	Final hearing level was better than 25 dB
Partial recovery	More than 15 dB of gain, final hearing 25–45 dB
Slight improvement	More than 15 dB of gain, final hearing poorer than 45 dB
No improvement	Less than 15 dB of gain or final hearing poorer than 75 dB

**Table 3:** General characteristics of the study subjects.

Type	SSNHL (*n* = 239)	Control (*n* = 239)	*p*-Value
Age, year	52.3 ± 16.3	54.5 ± 15.0	0.144 ^1^
Sex, Male/Female	126/113	126/113	1.000 ^2^
Waist, cm	86.0 ± 10.8	80.9 ± 9.9	0.001 ^1^
Fasting glucose, mg/dL	142.1 ± 61.6	101.1 ± 23.4	0.001 ^1^
Triglyceride, mg/dL	120.7 ± 76.5	88.1 ± 56.0	0.001 ^1^
HDL-C, mg/dL	52.2 ± 11.5	50.4 ± 12.1	0.110 ^1^
Systolic BP, mmHg	120.0 ± 12.7	117.5 ± 13.2	0.042 ^1^
Diastolic BP, mmHg	74.2 ± 8.2	72.8 ± 8.9	0.079 ^1^
Hypertension, *n* (%)	64 (26.9)	47 (19.7)	0.066 ^2^
Diabetes mellitus, *n* (%)	42 (17.6)	27 (11.3)	0.048 ^2^
Side, right/left	127/112		
**Outcome, *n* (%)**			
Complete recovery	61 (25.5)		
Partial recovery	42 (17.6)		
Slight improvement	50 (20.9)		
No recovery	86 (36.0)		

Abbreviation: HDL-C, high-density lipoprotein cholesterol. ^1^ Independent *t*-test. ^2^ *Chi*-square test.

**Table 4:** Prevalence among subjects of the components of metabolic syndrome.

Type	SSNHL (*n* = 239)	Control (*n* = 239)	*p*-Value	aOR ^1^ (95% CI)
Waist (male ≥ 90 cm, Female ≥ 80 cm), *n* (%)	122 (51.0)	89 (21.0)	0.001 ^2^	
Triglyceride ≥ 150 mg/dL	43 (18.0)	55 (17.7)	0.847 ^2^	
HDL-C (male < 40, Female < 50 mg/dL), *n* (%)	67 (28.0)	81 (15.0)	0.069 ^2^	
Fasting glucose ≥ 100 mg/dL or diabetic medication, *n* (%)	192 (80.8)	104 (36.8)	0.001 ^2^	
Blood pressure ≥ 130/85 mmHg or antihypertensive drug, *n* (%)	79 (33.1)	56 (27.6)	0.043 ^2^	
Metabolic syndrome, *n* (%)	99 (41.4)	42 (19.3)	0.001 ^3^	4.30 (1.98–9.33)

Abbreviation: SSNHL, sudden sensorineural hearing loss; HDL-C, high-density lipoprotein cholesterol; aOR, adjusted odds ration; CI, confidence interval. ^1^ Waist, glucose, triglyceride, systolic blood pressure, and DM adjusted. ^2^ *Chi*-square test. ^3^ Multivariate logistic regression analysis

**Table 5:** Base characteristics of the response and non-response groups.

Type	Response Group (*n* = 153)	Non-Response Group (*n* = 86)	*p*-Value
Age, year	49.7 ± 15.8	56.8 ± 16.6	0.001 ^1^
Sex, male/female	89/64	37/49	0.024 ^2^
Side, right/left	89/64	38/48	0.038 ^2^
Duration between onset to visit, days	3.7 ± 3.9	6.1 ± 6.9	0.001 ^1^
Hypertension, *n* (%)	13 (8.5)	9 (10.5)	0.613 ^2^
Diabetes mellitus, *n* (%)	25 (16.3)	17 (19.8)	0.504 ^2^
Dyslipidemia, *n* (%)	13 (8.5)	9 (10.5)	0.613 ^2^
Height, cm	164.1 ± 9.5	159.7 ± 10.7	0.002 ^1^
Weight, kg	66.5 ± 13.3	63.1 ± 14.3	0.069 ^1^
Hip, cm	95.5 ± 9.2	95.8 ± 7.6	0.831 ^1^
Vertigo, *n* (%)	42 (27.5)	36 (41.9)	0.023 ^2^
Tinnitus, *n* (%)	130 (85.0)	86 (100.0)	0.206 ^2^

Abbreviation: ^1^ Independent *t*-test. ^2^ *Chi*-square test.

**Table 6:** Factors that influence treatment outcome of sudden sensorineural hearing loss.

Type	Response Group (*n* = 153)	Non-Response Group (*n* = 86)	*p*-Value
Metabolic syndrome, *n* (%)	52 (34.0)	47 (54.7)	0.002 ^2^
Criteria I (waist), *n* (%)	76 (49.7)	46 (53.5)	0.561 ^2^
Criteria II (TG), *n* (%)	25 (16.3)	18 (20.9)	0.674 ^2^
Criteria III (HDL-C), *n* (%)	32 (20.9)	35 (40.7)	0.005 ^2^
Criteria IV (glucose), *n* (%)	117 (76.5)	76 (88.4)	0.073 ^2^
Criteria V (BP), *n* (%)	47 (30.7)	32 (37.2)	0.592 ^2^
Initial hearing threshold (dB)	71.4 ± 20.9	78.5 ± 24.9	0.019 ^1^
Final hearing threshold (dB)	34.3 ± 21.5	75.6 ± 26.9	0.001 ^1^
Hearing loss pattern			0.004 ^2^
Low tone	18 (11.8)	5 (5.8)	
Hight tone	23 (15.0)	22 (25.6)	
Flat	88 (57.5)	34 (39.5)	
Total	24 (15.7)	25 (29.1)	
Hearing loss severity			0.006 ^2^
25–40 dB	10 (6.5)	2 (2.3)	
41–70 dB	64 (41.8)	36 (41.9)	
71–90 dB	51 (33.3)	17 (19.8)	
>90 dB	28 (18.3)	31 (36.0)	

Abbreviation: BP, blood pressure; TG, triglyceride; HDL-C, high-density lipoprotein cholesterol. ^1^ Independent *t*-test. ^2^ *Chi*-square test.

**Table 7:** Multivariate logistic regression analysis of the hearing outcomes.

Type	Response (*n* = 153)	Non- Response (*n* = 86)	aOR ^1^	95% CI	*p*-Value
Height, cm	164.1 ± 9.5	159.7 ± 10.7	0.965	0.936–0.994	0.017
Duration, days	3.7 ± 3.9	6.1 ± 6.9	1.102	0.033–1.175	0.003
Metabolic syndrome					
No	101 (72.1)	39 (45.3)	1 (ref)		
Yes	52 (34.0)	47 (54.7)	2.182	1.211–3.932	0.009
Hearing loss pattern, *n* (%)					
Low	18 (11.8)	5 (5.8)	1 (ref)		
High	23 (15.0)	22 (25.6)	4.100		0.029
Flat	88 (57.5)	34 (39.5)	1.503		0.490
Total	24 (15.7)	25 (29.1)	4.600	1.320–16.026	0.017

Abbreviation: aOR, adjusted odds ratio; ref, reference category; CI, confidence interval. ^1^ Adjusted for age, sex, side, vertigo, hearing loss severity, and criteria III.
